# Bridging research and practice: a pilot trial of an adapted social skills program for autism in public services 

**DOI:** 10.3389/fpsyt.2025.1610221

**Published:** 2025-12-04

**Authors:** Maria A. Mairena, Carlota Alcover, Eulàlia Arias-Pujol, María Díez-Juan, Marcela Mezzatesta Gava, Neus Elias, Ares Sentenach, Maria Elias, Eulàlia Piera, Mireia González, Marjorie Solomon, Aritz Aranbarri

**Affiliations:** 1 Child and Adolescent Mental Health Research Group, Institut de Recerca Sant Joan de Déu (IRSJD), Esplugues de Llobregat, Spain; 2 Department of Child and Adolescent Mental Health, Hospital Sant Joan de Déu, Esplugues de Llobregat, Spain; 3 Social Determinants and Health Education Research Group (SDHEd), Hospital del Mar Research Institute, Barcelona, Balearic Islands, Spain; 4 Hospital del Mar Nursing School (ESIHMar), Universitat Pompeu Fabra-Affiliated, Barcelona, Balearic Islands, Spain; 5 Faculty of Psychology, Education and Sports Sciences Blanquerna, Research Group of Couple and Family (GRPF), Barcelona, Catalonia, Spain; 6 Department of Psychiatry and Behavioral Sciences and the Medical Investigation of Neurodevelopmental Disorders (MIND) Institute University of California (UC) Davis, Sacramento, CA, United States

**Keywords:** autism spectrum disorders, social skills, public mental health, adolescence, child

## Abstract

**Background:**

Autism Spectrum Disorder (ASD) is marked by significant challenges in social communication and interaction, often accompanied by comorbid neurodevelopmental and mental health conditions such as anxiety and depression. These social difficulties can interfere with the development of pragmatic language use, social problem-solving abilities and quality of life. Social skills interventions have shown promising results in addressing these challenges, yet there is a need to adapt such programs for broader, publicly funded settings to enhance accessibility and effectiveness.

**Objective:**

This study reports on a randomized, waitlist-controlled pilot efficacy trial evaluating an adapted version of the Social Adjustment Enhancement Intervention Program implemented in a university-affiliated public mental health hospital.

**Methods:**

A 10-session group-based intervention was delivered to 79 autistic participants aged 8–17 years. Social behaviors were assessed before and after the intervention using structured observational methodology, and comorbid internalizing symptoms were measured via parent-report standardized questionnaires.

**Results:**

Significant improvements were observed in the subgroup of children and in participants with higher verbal IQ, particularly in eye contact and functional communication, as measured through observational methodology. Additionally, a reduction in internalizing symptoms was found among children in the experimental group. In the full sample, no statistically significant effects were observed, although trends in the same direction were noted.

**Conclusions:**

Findings support the feasibility and pilot evidence of intervention effects of this adapted social skills program, particularly for younger autistic individuals and those with higher verbal abilities. The results underscore the importance of tailoring interventions to age and cognitive profiles, and highlight the value of accessible, evidence-based approaches in public mental health systems. Further research is needed to optimize intervention design and evaluate long-term outcomes.

## Introduction

Autism is characterized by difficulties in social communication and interaction and the presence of repetitive patterns of behaviors, interests, or activities (1). The incidence of autism continues to increase, both at the global and local levels. According to the latest data in the US, 1 in 36 children at 8 years old meet the criteria for an autism diagnosis (3). In addition to autistic traits, approximately 82% of autistic children present with coexisting neurodevelopmental conditions (e.g., language difficulties, attention deficit–hyperactivity symptoms), intellectual developmental delay (4–7), sensory dysregulation or another mental condition(e.g., anxiety, obsessive-compulsive symptoms and/or oppositional defiant behaviors; (8, 61, 66) that affects their behavior and overall functioning.

Differences in social communication and interaction may interfere with the pragmatic use of language, social skills development, conversational abilities (9, 10), social problem-solving (11, 12) and development of theory of mind (ToM). These social challenges may reduce their quality of life.

In addition, symptoms of anxiety and depression are also common among individuals with social difficulties (13). This symptomatology is sometimes a consequence of different sensory integration, problems interacting with others, difficulties in understanding demands from the environment (12, 14), or the awareness of their own challenges (15). Moreover, anxiety and depression symptomatology might be related to behavioral difficulties (16), which may also interfere with their interactions with other peers.

### Social skills intervention programs

Regarding treatment, evidence-based practices recommended for autistic children and adolescents include social skills training (17), frequently implemented in group format in school environments (18). Various social skills programs have been proposed, such as PEERS (19), Think Social! (20), Social Skills Training (21), Navigating the Social World (22) and Social Adjustment Enhancement Intervention (2).

Specific social skills training programs have effectively increased social competence, peer interaction, and functional behaviors while decreasing time alone and repetitive behaviors (23, 24). Research has also found a decrease in overall anxiety (25) and social anxiety (12) after participation in social skills intervention programs. However, some authors (e.g., 26) have suggested that social skills training may also have the potential to exacerbate anxiety, depending on the individual’s mental health profile. Similarly, several studies (e.g., 27, 28) have raised concerns about the potential negative impacts of social skills interventions on autistic individuals. These studies highlight that while such interventions might help individuals conform to socially appropriate behaviors, they can also lead to increased anxiety and emotional distress. Therefore, researchers such as Antshel et al. (29) propose to assess the influence of anxiety on social skill treatment outcomes for children with autism and to investigate whether anxiety would moderate treatment efficacy.

Building on these considerations, certain factors, such as intelligence quotient and age (30), may influence treatment response, but these relationships are unclear. The literature highlights the effectiveness of social skills intervention programs for participants with an IQ above 70 (31), noting explicit improvement in comorbid depression symptoms (2). A systematic review concluded that better cognitive functioning and verbal ability are related to a greater treatment response overall (23). Regarding age, studies suggest more positive results for adolescents than children, with particularly favorable outcomes reported among adolescent females (32). However, there is still considerable controversy about the role of participants’ age in treatment outcomes.

In addition to individual characteristics, contextual and structural aspects of the intervention, such as setting and duration, may also impact treatment outcomes. There have been relatively few studies examining low-intensity social skills programs consisting of limited sessions (typically 10–15 sessions). When implemented in school settings, several studies have demonstrated improvements in participants’ functioning within the school environment (25, 33), enhanced ability to perceive anger (34), reductions in social anxiety symptomatology (12), and an overall improvement in social skills among children and adolescents (35). However, there remains a significant need to evaluate the efficacy of adapted manualized interventions delivered in lower-intensity formats, particularly those designed to meet the needs and constraints of publicly funded community services.

### Measuring change

In terms of assessment, greater consensus is needed regarding the most sensitive method to evaluate changes related to treatment response. Standardized questionnaires may not reflect changes in social skills observed during sessions, but they are helpful to assess changes in comorbidity (23, 36). In response to these limitations, researchers have proposed integrating data obtained from questionnaires (quantitative measures) with observational methodologies (qualitative measures) to analyze spontaneous behaviors that occur in response to various social situations and prompts (37). Although further research is needed, observational methodology is increasingly utilized in autism research (38, 39), providing new methods that may contribute to more sensitively capturing the subtle behaviors occurring in social skills development. Along this line, Dean and Chang (40) conducted a systematic review of school-based social skills interventions, specifically examining studies that used observational measures. According to their review, all studies reported improvements in social behaviors, while observation protocols detected more subtle changes.

Recent work by Moody et al. (41) supports the integration of multimodal assessment tools, including behavioral coding systems and ecological momentary assessment, to improve the accuracy of treatment response evaluation in autistic populations. These approaches offer promising directions for future research and clinical practice. The authors also emphasize the importance of moving beyond traditional informant-report measures to include observational and culturally sensitive methods that better reflect the diversity of autistic experiences.

### Present study

This study was conducted as a randomized, waitlist-controlled pilot efficacy trial to evaluate an adapted version of the Social Adjustment Enhancement Intervention (SAEI) (2), designed for transferability to publicly funded services.

The first step of this study was to develop an adaptation of the social skills training program proposed by Solomon et al. (2): Social Adjustment Enhancement Intervention of the University of California, Davis MIND Institute, a program for autistic children and adolescents as well as those with related communication challenges. The program was designed as a 20-session group program where therapists promote natural and spontaneous social interactions, including reciprocal conversation, cooperative play, and a sense of humor. Previous research found positive results, such as increased socially oriented responding vocalizations and decreased non-social vocalizations (e.g., talking alone) after the intervention (9).

For this study, the research team developed a shorter version of the program to make it feasible in a publicly funded university hospital mental health setting, to facilitate future implementation in community-based services.

Once the program was adapted, the primary goal was to conduct a randomized waitlist-controlled trial to evaluate the effect of our adapted social skills program on autistic children and adolescents. Both observational methodologies and standardized questionnaires were used to assess treatment response, allowing for the examination of real-time social behaviors as well as broader symptom profiles. The study sought to capture the nuanced and immediate changes in social interactions resulting from the intervention, providing a comprehensive understanding of its impact on participants’ social competencies.

Based on this goal, the following research questions were proposed: (1) Do participants in the intervention group demonstrate increased social behaviors (e.g., eye contact, smiling, functional communication, and verbal social interaction) following the intervention, compared to the control group? It was hypothesized that participants in the intervention group would exhibit a higher number of social behaviors in a group situation after receiving the program than the control group (2, 9, 62) (2). What are the differential effects of social skills interventions on anxiety and affective symptoms in autistic individuals, considering both improvements and potential exacerbations? Based on previous research on this program (2), it was hypothesized that participants in the intervention group would show no negative impact on their anxiety or affective problems and might even show improvement in comorbid symptoms after the intervention (12, 25). An exploratory question was also examined: (3) Do treatment outcomes vary by verbal intelligence quotient (IQ) and age group? This question was intended to explore whether the intervention produces differential effects for children versus adolescents, and whether individuals with a verbal IQ above 90 show a more favorable response compared to the full sample.

## Method

### Participants

Psychologists and psychiatrists recruited participants from the Autism unit of a public hospital after being evaluated by highly trained, specialized clinicians in autism. Inclusion criteria were: age range between 8 and 17 years old, a confirmed clinical diagnosis of autism according to DSM 5 (1), meeting the cut-off of Autism at the Autism Diagnostic Observation Schedule-2 (ADOS-2, 42), and a standard range of Verbal Comprehension (Verbal IQ with a cutoff of 80, measured by the Wechsler Intelligence Scale for Children - IV, 2007, 2014). Participants with severe behavioral problems, several mental conditions such as schizophrenia, or intellectual functioning below an IQ score of 80 were excluded. Participants were excluded if they presented intellectual functioning below an IQ score of 80 or a diagnosis of several mental disorders (e.g., schizophrenia, bipolar disorder, conduct disorder or oppositional behavior disorder). All mental health diagnoses were established according to DSM-5 criteria via clinical interview administered by licensed clinical psychologists.

**Figure 1:**
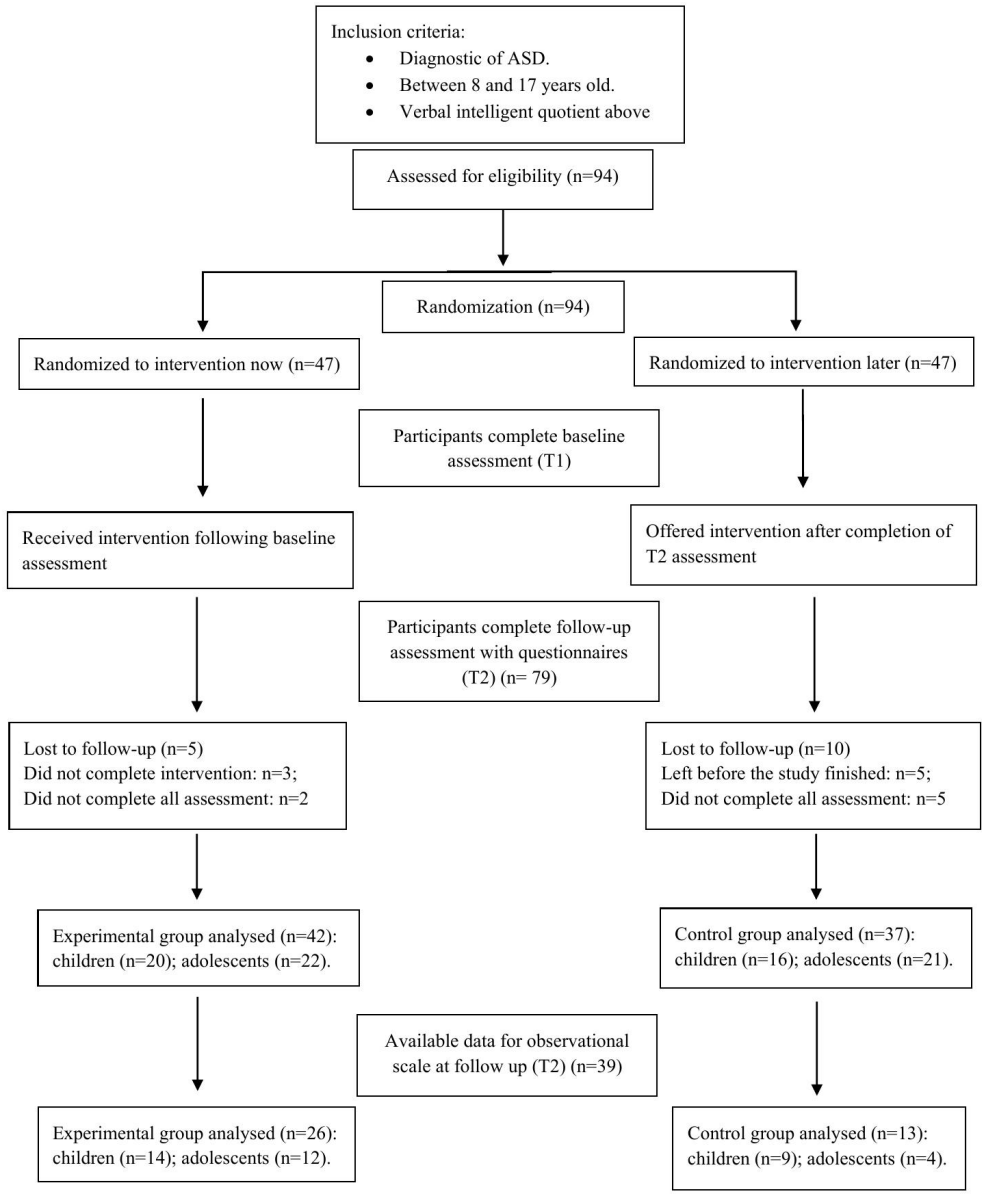
Flowchart of participant recruitment and retention.

A total sample of 94 participants was divided into two age groups: children (8 to 12 years old, n=36) and adolescents (13 to 17 years old, n=43). Subjects were paired according to age, sex, and verbal IQ; then each pair was randomized between the two groups (experimental group and waiting-list control group) using R software (R Foundation for Statistical Computing; Vienna, Austria) with a fixed random seed (see the Consort Diagram in **Figure 1**).

Although the full randomized controlled trial (RCT) sample included 94 autistic children and adolescents (i.e., 36 children and 43 adolescents) who completed the social skills intervention, only a subsample of 39 participants met the criteria for inclusion in the observational methodology analysis. These criteria included: 1) attending the specific recorded sessions (i.e., sessions 2 and 10); 2) availability of high-quality video recordings of the sessions, which were required for reliable coding; availability of video recordings with sufficient duration and appropriate camera angles to reliably code the target behaviors, based on audiovisual characteristics (see **Figure 1**), (3) the presence of observable and analyzable social interaction moments within the group setting. The observational methodology was applied to this subsample to ensure consistent and valid measurement of spontaneous social behaviors. Participants in the full sample were divided into two developmental groups (children and adolescents) and an exploratory analysis was conducted to examine whether those with a verbal IQ above 90 showed a more favorable treatment response compared to the overall sample.

### Design

A randomized, waitlist-controlled pilot efficacy trial was conducted between 2017 and 2019 to evaluate the impact of the adapted intervention on social behaviors, internalizing symptoms, and anxiety. Participants were randomized by age, gender, and verbal IQ. The intervention condition was compared at two time points: baseline (T1) and three months later (T2). During these three months between assessments, the experimental group received our adapted social skills intervention program, which consisted of 10 sessions delivered once per week, while the waiting-list control group received the intervention after the T2 assessment.

### Intervention description

The social skills intervention program was a consented adaptation of the Social Adjustment Enhancement Intervention of the UC Davis MIND Institute (2). While the original program included 20 sessions and a parent component, the adapted version was reduced to 10 group sessions to improve feasibility in a publicly funded mental health setting. The parent component was removed. Prior community research (e.g., 35, 43) has highlighted the challenges of implementing intensive, evidence-based interventions in these settings, where resource limitations and long waiting lists often necessitate shorter programs that can serve more children. However, However, these adaptations must be rigorously tested before broader community implementation. Importantly, the core components of the original program were preserved.

The original program comprised two modules of 10 sessions: The first module includes topics related to emotion recognition, stress management, and conversational skills, while the second module is centered on social problem-solving and developing a video. The adapted 10-session version retained six sessions on emotional competence (e.g., empathy, recognition, and regulation), two sessions on non-verbal communication and conversation, and one on problem-solving. Problem-solving was also addressed indirectly through real-time support during free play, when challenges naturally emerged.

Each session lasted 90 minutes and was delivered in a group format designed to foster internal motivation for social learning, with individualized goals, cognitive-behavioral strategies, role-play, peer modeling, and clinician-supported opportunities for building friendships. Sessions followed a consistent structure: greeting, free play (unstructured peer interaction), a didactic activity (focused on social competence), joke-telling, and closing activities, which included optional homework (“social experiment for the week”). Didactic content addressed key areas of social competence such as empathy, expressing feelings, and resolving social conflicts.

In addition to following the structure proposed by Solomon et al. (2), clinicians incorporated strategies based on Theory of Mind and social cognition (44). Each session aimed to support the development of social skills frequently impacted in autistic youth (see **Table 1** for general structure and **Table 2** for session-specific content).

**Table 1:** General structure of the sessions.

Parts of the session	Explanation of the content
Introduction	Every session starts with an introduction to the topic of the session. During this introduction, participants have space to talk and share thoughts or problems about the week.
Free time play	Participants have 15 minutes to choose and play one of the games that are given by the therapist (UNO, Who’s who? Mikado and animals, among others). It is an unstructured time where they are not guided by the therapists. Children can decide if they want to play, talk with others, or just remain alone. This is the most spontaneous part of the session. Therapists may intervene to discreetly encourage some interactions and prevent unexpected behaviors (e.g. insulting).
Didactic activity	This is a structured part, where the therapists work on different topics, such as friendship, solving problems, empathy, or emotions. All topics proposed are related to difficulties with social competence.
Closing	Every session closes with a structured joke telling time. At the end, participants get a “social experiment” (optional homework related to the lesson topic worked on didactic session).

Based on 2.

**Table 2:** Specific contents of each session.

Number of sessions	Subject of the didactic activity
Session 1	Didactic activity: “The three steps of empathy”: recognizing others’ feelings and giving and appropriate response.
Session 2	Didactic activity: “Review personal objectives and practice empathy”: role-play to practice empathy.
Session 3	Didactic activity: “Emotions”: knowing your feelings; recognizing signals in your body.
Session 4	Didactic activity: “Practice emotional recognition”: knowing your feelings; connecting situations to feelings.
Session 5	Didactic activity: “Stress and anxiety: possible strategies to feel better”: emphasis on uncomfortable feelings and stress management techniques.
Session 6	Didactic activity: “Stress and anxiety: possible strategies to feel better”: emphasis on uncomfortable feelings and stress management techniques.
Session 7	Didactic activity: “Interests and conversation”: appropriate topics, rules for conversations.
Session 8	Didactic activity: “Nonverbal communication and conversation”: gestures, facial expression, posture, tone of voice.
Session 9	Didactic activity: “Solving problems”: identifying the problem, generating alternative solutions.
Session 10	Closing group. Talk about feelings and explain (if they want) what they learned during all the sessions.

Adapted from Solomon et al. (2).

### Measurements

#### Clinical characterization: ASD features and intellectual abilities

The Autism Diagnostic Observation Schedule (ADOS-2; 42) was administered by highly trained, specialized clinicians. All assessors were trained through the US or Spain’s official advanced ADOS-2 training program. Intellectual abilities and verbal comprehension were measured with the Wechsler Intelligence Scale for Children and Adolescents: Fourth (45) or Fifth Edition (46).

#### Measures of change in response to treatment: Primary outcome: *Ad hoc* observational instrument to code social behaviors

Treatment-targeted subtle social behaviors were measured through an observational instrument that allowed the definition and code of specific behaviors during a free playtime session (14 minutes) at baseline (T1) and three months later (T2). The experimental group received the social skills intervention program between these two assessment time points.

The observational instrument (39) was based on both Bauminger’s scale (2002), and the definitions of social difficulties provided by the ADOS-2 and the ADI-R (42, 47). Bauminger’s instrument has also been used in previous research assessing a social skills intervention program (9). Through this observational instrument, we aimed to quantify occurrences of target social behaviors: eye contact, smiling, functional communication, and social-verbal communication (see more detailed information in **Table 3**).

**Table 3:** Category systems of our observational instrument (adapted from 39).

Behavior	Description
Eye contact (EC)	Participant looks into the eyes of another child during a conversation or while playing.
Smile (SMIL)	Participant smiles to another in an interaction
Social verbal communication (SOVERC)	Participant approaches another child with a social intention.
Functional communication (FUNC)	Participant approaches or responds to another child with an intention to fulfill his/her own needs, and with no social intention or just to express something related to the game, not with a social intention.

#### Secondary outcomes: Child Behavior Checklist (CBCL/6-18)

The CBCL (48) is a parent-completed questionnaire that allows to assess behaviors and emotional problems in subjects aged one year six months to five years six months old and six to eighteen years old. Both internalizing behaviors (e.g., anxiety, depression, etc.) and externalizing behaviors (e.g., aggression, hyperactivity, etc.) are included. The present study focuses on three subscales: Anxiety, Affective Problems and Internalizing Problems.

#### The Spence Children’s Anxiety Scale (SCAS)

The SCAS (49, 64) is a measure of anxiety disorders in childhood and adolescence. There is a self-report and a parent-report version. It consists of 44 items that assess separation anxiety, obsessive-compulsive disorder, panic, social phobia, generalized anxiety, etc. It is evaluated through a Likert scale, with a maximum score of 114, an average of 57, and a minimum score of 0. The present study focuses on subscales of the parent-report version: social phobia and generalized anxiety.

### Procedures

All procedures followed the Declaration of Helsinki 2000 and our hospital’s Ethical Committee for Clinical Research standards. This study was registered in the ClinicalTrials.gov database (Identifier: NCT05713162). Parents of eligible participants were invited to attend an informative session in which the study was presented prior to recruitment. Afterwards, parents provided signed consent, and informed assent was obtained from the child or adolescent.

The study was conducted over three years (i.e., 2017, 2018, and 2019). Each year, the team ran an experimental group of children and another group of adolescents. Treatment sessions were delivered from January to April, and each treatment group consisted of 7 to 9 participants who received the same intervention conditions.

The intervention was delivered to a group therapy room within a pediatric public hospital (review further details in the *Intervention Description* section). After approval by Solomon and colleagues, the original program materials were translated, adapted, and culturally tailored for local use. A senior psychologist from our department—previously involved in implementing the original intervention at the University of California, Davis, MIND Institute (US), provided training to ensure consistency with the original model. To ensure fidelity to the original program, the clinical team reviewed the implementation protocol and completed a session-specific checklist at the end of each session.

To assess treatment response in social behaviors, researchers’ video-recorded the 14-minute free play period from the second (T1) and last treatment session (T2). The first session was excluded to avoid potential bias in social behavior due to participants meeting for the first time. Free play occurred in an unstructured format, where participants could interact using various games provided or choose to play alone. Therapists and co-therapists interacted only as needed to prompt or observe spontaneous social behaviors, using this segment to track progress. Two cameras were positioned at different angles to record sessions discreetly, following all ethical guidelines. Participants were informed about the purpose, location, and use of the video recordings for research. Two trained psychologists coded all targeted social behaviors. They went through a reliability process in order to assure the quality of the data. Raters discussed codes when there was disagreement. Video coding was performed using LINCE software, a validated multiplatform tool (50). To assess changes in comorbid anxiety and affective symptoms, parents completed standardized questionnaires (CBCL and SCAS) pre- and post-intervention.

### Data analysis

Data were analyzed with the Statistical Package for the Social Sciences (SPSS v23.0) (60). Participants were characterized through descriptive analyses. To evaluate whether there was a treatment effect on the number of social behaviors, a 2 X 2 (time x treatment group) ANOVA was conducted for the target observational scale outcomes: eye contact, smile, functional communication, and verbal social communication.

The second objective aimed to evaluate whether participants in the intervention group (experimental group) showed lower levels of internalizing symptomatology at the post-treatment assessment than participants in the control group (waitlist group). This analysis also helped researchers ensure no unwanted side effects of the intervention. To test this, a 2 X 2 (time x treatment group) ANOVA was conducted for specific subscales of the CBCL (anxiety problems, affective problems, internalizing problems) and the parent-report SCAS (social phobia and generalized anxiety) questionnaires.

The last research question asked whether there were differences in the treatment effect for all outcomes based on the group age (children and adolescents) and verbal IQ (VIQ). First, 2 X 2 X 2 (time x treatment group x age group) ANOVA models were performed. Afterwards, researchers selected the sample of children and evaluated significant changes in the experimental group versus the waitlist control group (time x treatment group). The same analyses were replicated for adolescents.

Finally, the study attempted to evaluate treatment response associated with participants’ verbal IQ. To test this question, participants were selected based on their VIQ, using a score of 90 as the cut-off and selection criteria. This allowed detect differences in the treatment responses of participants with a higher VIQ compared to the results obtained with the total sample. For this purpose, researchers conducted a 2 X 2 ANOVA (time x treatment group) within the sample with an IQ over 90 (n=60).

All statistical tests were performed with a bilateral contrast, and the significance level was set at 0.05.

Although a multiple ANOVAs was conducted to test hypotheses concerning social behaviors and internalizing symptoms, no formal adjustment for multiple comparisons was applied.

This decision was made because of the pilot nature of this study and because the analyses were hypothesis driven.

## Results

### Participant features and acceptability

All participants in the total sample (N = 79; 36 children and 43 adolescents) received the entire treatment program. They were 82.3% male (mean age: 11.92; mean verbal IQ: 105.73). Participants in the sub-sample analyzed for observational data (n=39) were 84.16% male (mean age: 11.27; mean verbal IQ: 104.08). Normality and statistical tests were performed to assess whether there were differences between the participants who only had questionnaires as a measure and those who had questionnaires and videos. There were no differences between groups. Descriptive results are shown in **Table 4**.

**Table 4:** Descriptive statistics of participants per group.

Descriptive characteristics	Total sample (N = 79)			Sub-sample for observational data (n=39)		
	Experimental group	Control group	Total	Experimental group	Control group	Total
Participants per group	42	37	79	26	13	39
Number of children	20	16	36	14	9	23
Number of adolescents	22	21	43	12	4	16
Age of children Mean (SD)	9.37 (1.165)	9.53 (1.302)	9.44 (1.211)	9.50 (1.160)	9.75 (1.282)	9.59 (1.182)
Age of adolescents Mean (SD)	15.09 (12.705)	13.86 (1.062)	13.88 (1.074)	14.08 (1.084)	13.25 (0.500)	13.88 (1.025)
Sex	33 males 9 females	32 males 5 females	65 males 14 females	21 males 5 females	12 males 1 female	33 males 6 females
Verbal IQ Mean (SD)	107.50 (14.094)	103.60 (16.457)	105.73 (15.136)	104.84 (12.099)	102.62 (17.476)	104.08 (13.970)

Participants, parents, and clinicians reported a high level of verbal acceptability, but no specific rating scale was administered. Clinicians participating in the study showed great satisfaction, as the training did not require expensive materials or specific equipment. Free playtime was highly motivating for participants, especially when they found common interests.

### Effects on social behavior

The first research question was whether there were changes in the number of social behaviors in the intervention group between baseline and follow-up. **Table 5** shows the means and standard deviations at baseline and follow-up for the experimental and control groups and the ANOVA statistics. Results from the ANOVA did not show significant differences between groups after the intervention; however, the experimental group showed a marginally significant increase in *eye contact* (F = 3.17; *p* = .083; effect size = 0.079) and the control group demonstrated a decrease in functional communication (*M_Pre_*= 16.08, *M_Post_*= 11.31). Although these differences did not reach conventional significance thresholds, they may suggest trends worth exploring in future studies. No differences suggesting potential effects were observed for smiling or social verbal communication (see **Table 5**).

**Table 5:** Means, standard deviations and ANOVA statistics for the observational instrument (n=39).

Group	Experimental group (n=26)		Control group (n=13)		Statistics values		
Category/Session	T1	T2	T1	T2	F test	P value	Effect size
	M (SD)	M (SD)	M (SD)	M (SD)			
Eye contact	1.81 (3.43)	3.12 (4.26)	2.08 (2.87)	0.92 (1.12)	3.17	0.083	0.079
Smile	1.46 (3.97)	2.08 (3.5)	1.31 (3.04)	0.77 (0.93)	0.86	0.359	0.023
Social Verbal communication	2.69 (5.3)	2.46 (4.25)	1.08 (1.55)	1.08 (1.98)	0.02	0.888	0.001
Functional communication	14.23 (9.52)	15.69 (10.64)	16.08 (12.31)	11.31 (8.4)	3.79	0.059	0.093

Estimates represent the interaction between time and the intervention group.

### Effects on comorbid internalizing symptomatology

The second research question was whether there were significant changes in comorbid internalizing symptomatology after the intervention, considering both the intervention’s beneficial and potentially harmful effects. **Table 6** shows the means and standard deviations of baseline and follow-up for the experimental and control groups and the ANOVA statistics. Results from the ANOVA showed a small, non-significant decrease in the subscale affective problems from the CBCL (F = 2.99; *p* = .09; effect size = 0.041) in the experimental group. This subscale includes problems such as fatigue, apathy, sadness, and inferiority complex, among others. Other target scales from the CBCL and the Spence Children’s Anxiety Scale did not show any significant or marginal differences (see **Table 6**), suggesting no significant negative side effects of the intervention.

**Table 6:** Means, standard deviations and ANOVA statistics for outcomes of questionnaires (CBCL and SCAS; n=79).

Group	Experimental group (n=36)		Control group (n=43)		Statistics values		
Category/Session	T1	T2	T1	T2	F test	p value	Effect size
	M (SD)	M (SD)	M (SD)	M (SD)			
CBCL dimensions							
Anxiety problems	4.33 (2.33)	3.81 (1.95)	5.72 (2.72)	5.14 (2.8)	0.01	0.9	0.00
Affective problems	7.22 (4.32)	6.14 (4.33)	7.42 (3.62)	7.53 (3.4)	2.99	0.09	0.04
Internalizing problems	18.58 (7.77)	16.06 (7.42)	20.94 (9.1)	20.14 (8.5)	1.66	0.2	0.02
SCAS dimensions							
Social phobia	6.09 (4.22)	5.41 (3.37)	6.38 (3.48)	5.59 (3.23)	0.23	0.88	0.00
Generalized anxiety	7.62 (3.55)	7.18 (3.15)	7.5 (3.53)	7.06 (3.69)	0.0	0.99	0.00
Social phobia parents report	5.59 (2.77)	5.46 (2.65)	6.76 (3.21)	5.94 (2.99)	1.39	0.24	0.02
Generalized anxiety parents report	4.68 (2.12)	5.11 (2.47)	6.54 (3.32)	6.03 (3.21)	2.4	0.13	0.03

Estimates represent the interaction between time and the intervention group. CBCL, Child Behavior Checklist (48); SCAS, The Spence Children’s Anxiety Scale (49).

### Treatment response based on verbal IQ and age

Based on the previous literature, treatment responses based on age and verbal IQ were examined, replicating analyses on the children group, the adolescent group and the higher IQ subgroup. The subgroup of 23 children (experimental group=14, control group=9) showed significant differences between groups after the intervention in the frequency of *eye contact* (F = 4.64, *p* = .043; effect size = 0.181) and *functional communication* (F = 5.09, *p* = .035; effect size = 0.195). The experimental group showed an increase in the frequency of eye contact (*M_Pre_*= 0.29, *M_Post_*= 1.86), while the control group diminished the frequency of eye contact (*M_Pre_*= 1.89, *M_Post_*= 0.78). Regarding functional communication, the experimental group showed a slight increase after the intervention (*M_Pre_*= 13.64, *M_Post_*= 14.93), whereas the control group demonstrated a decrease (*M_Pre_*= 19, *M_Post_*= 12.33). Moreover, a difference that approached but did not reach statistical significance between groups was observed for *smiles* (F = 3.73, *p* = .067). The experimental group showed an increase in the frequency of smiles (*M_Pre_*= 0.29, *M_Post_*= 1.29), while the control group exhibited a diminished number of smiles (*M_Pre_*= 1.78, *M_Post_*= 0.44). Results did not show differences in social-verbal communication in the group of children (see **Table 6**).

Results from questionnaires in the group of children revealed significant differences between groups after the intervention in the CBCL *internalizing* subscale (F = 6.09, *p* = .02; effect size = 0.16). The experimental group showed a decrease in the score of internalizing problems (*M_Pre_*= 16.17, *M_Post_*= 12.44), whereas the control group maintained a similar mean (*M_Pre_*= 17.75, *M_Post_*= 18.5). Additionally, a small, non-significant reduction was observed for the CBCL *affective problems* subscale (F = 3.08, *p* = .09) for the experimental group. No other CBCL or SCAS subscales showed notable differences between groups in the children subgroup, and none of the effects reached statistical significance.

In the subgroup of adolescents, no significant results were observed in either studied variable of the observational scale (n=16). For questionnaires (n=43), results from the ANOVA analyses showed significant differences between groups after the intervention in the parent report subscale of the SCAS *generalized anxiety* (F = 6.19, *p* = .02; effect size = 0.15). The experimental group showed an increase in generalized anxiety (*M_Pre_*= 4.33, *M_Post_*= 5.28).

Of the total sample (N = 79), 60 participants had a verbal IQ>90 (experimental group=35, control group=25). Results showed a significant difference between groups in *eye contact* (F = 4.52; p = .04; effect size = 0.148) and *functional communication* (F = 5.56; p = .03; effect size = 0.176). The experimental group showed an increase in the amount of eye contact (Children: *M_Pre_*= 0.18, *M_Post_*= 1.55; Adolescents: *M_Pre_*= 3.3, *M_Post_*= 5.3), while the control group showed a decrease (Children: *M_Pre_*= 2.13, *M_Post_*= 0.87; Adolescents: *M_Pre_*= 7, *M_Post_*= 2).

Regarding functional communication, results indicated an increase for the experimental group (Children: *M_Pre_*= 13, *M_Post_*= 14.18; Adolescents: *M_Pre_*= 15.1, *M_Post_*= 16.2), while the control group showed a decrease (Children: *M_Pre_*= 18.38, *M_Post_*= 13.5; Adolescents: *M_Pre_*= 32, *M_Post_*= 13).

No significant differences between groups after the intervention were observed in the results of the questionnaires in the group of participants with VIQ>90, showing no unwanted side effect of the intervention in this higher-functioning subgroup.

## Discussion

This study was designed as a pilot efficacy trial to evaluate the impact of an adapted version of the Social Adjustment Enhancement Intervention (2), developed for transferability to publicly funded services. The intervention was implemented in a university-affiliated pediatric hospital. Although the setting was not a community-based service, it operated under clinical care demands and resource limitations, with implementation carried out by a specialized autism team. Following prior community-oriented research (e.g., 35, 43), a shortened version of the program was developed and tested in a randomized, waitlist-controlled pilot trial with autistic children and adolescents. While the findings are promising, they should be interpreted within a feasibility framework and viewed as a foundation for future effectiveness research in community-based services.

The most robust finding of this study was a significant improvement in observed social behaviors—specifically eye contact and functional communication—in the subgroup of children who received the intervention. This supports the program’s potential to enhance core social interaction abilities in younger autistic individuals. Additionally, a significant reduction in internalizing symptoms was observed in this same subgroup, as measured by the CBCL, suggesting a possible emotional benefit in parallel to social gains. These findings are consistent with previous studies reporting improvements in both social behaviors and emotional functioning following social skills interventions in autistic populations (2, 9, 25). Moreover, they align with research suggesting that age may play a key role in moderating treatment effects in social skills programs (30, 51).

Similarly, participants with higher verbal IQ (VIQ > 90) also showed significant improvements in eye contact and functional communication. These findings are consistent with previous literature indicating that higher verbal and cognitive abilities may enhance response to social skills interventions (23). Taken together, these subgroup effects highlight the importance of tailoring interventions based on developmental and cognitive profiles to increase their effectiveness.

In contrast, no statistically significant improvements were observed in the full sample across the assessed social behaviors. However, a general pattern of increased eye contact and functional communication in the experimental group was noted, which may indicate an early signal of intervention effects requiring confirmation in larger, fully powered studies. The absence of broader effects in the total sample may be partly attributed to the limited sample size for the observational analysis and the reduced duration of the adapted program.

These results align with other studies that have found a significant increase in eye contact after a social skills intervention (52). Unlike our program, Bauminger (52) implemented their intervention over a more extended period (7 months) and at school settings, involving children, teachers, parents, and peers. These differences could have a meaningful impact on the effectiveness of the intervention. A longer duration of the program and an implementation in a natural setting (the school) and involving adults of reference (teachers, parents) could offer participants the opportunity to learn and practice how to interact with their peers in the natural environment where these behaviors occur (53).

Similarly, two independent studies that used the complete version of the original program (2, 9) found significant positive results in social behaviors. In these studies, a longer duration (20–22 weeks) and including peers without autism differentiated them from the present study. These factors are assumed to play a key role in enhancing a higher efficacy of the intervention, showing a higher consistency of measured social behaviors and facilitating the generalization of trained skills in patients’ natural contexts (54, 55). Another distinction between this study and previous investigations is the inclusion of intervention sessions involving parents. This can contribute not only to the generalizability and maintenance of the social skills learned in the intervention groups but also to increased opportunities for practicing these skills in natural settings. This, in turn, may strengthen their consolidation and frequency in everyday interactions and directly influence how often these behaviors are observed and coded as treatment response markers in clinical trials.

In addition to examining potential benefits, this study also incorporated a safety-focused perspective by evaluating possible unintended or adverse effects of the intervention—an approach not commonly included in community-adapted mental health programs. In this context, the observed increase in generalized anxiety within the experimental group of adolescents, as reported by parents through standardized questionnaires, constitutes an important finding. Although not a desired outcome, it reflects the ethical commitment to monitor both risks and benefits in the real-world implementation of social skills interventions. This result diverges from previous findings showing anxiety reductions in similar programs (e.g., 56, 59), as well as other group-based social interventions targeting social cognition and behavior (63) and raises critical questions about how adolescents with autism respond to brief, community-translatable formats of structured interventions.

The relationship between anxiety and autism-related social difficulties is complex, and more research is needed to understand its association. Certain previous studies using social skills interventions for adolescents with autism did not find a decrease in anxiety symptoms (57). A possible reason is that participants may become more aware of their limitations through the program. Moreover, anxiety has been associated with higher social challenges in autism (58), and the adolescent group in this study showed less improvement in social behaviors than children, which might reflect more complexity in the intersection between social skills and anxiety. It is also possible that the timing of the treatment response measurement, based on the frequency of target social behaviors, was affected by the interaction with higher anxiety levels, which appeared to increase at the end of the adapted program. An alternative explanation for this result could rely on the program duration. A shorter program may not provide participants with sufficient time to develop the targeted social skills or to develop coping strategies effectively that may help manage their anxiety in social group interactions. Consequently, adolescents may experience heightened anxiety levels as they navigate unfamiliar social situations or encounter new challenges introduced by the program. This suggests a nuanced relationship between program duration and anxiety levels. While shorter programs may trigger initial increases in anxiety, longer interventions may offer opportunities for adaptation and eventual symptom reduction. The present findings indicate that brief social skills interventions may carry a risk of unintended side effects, such as increased anxiety in specific subgroups. This underscores the importance of further research to ensure that social skills programs do not inadvertently exacerbate anxiety symptoms. In contrast, participating children showed significant improvements in both anxiety and affective symptoms.

Overall, professionals must ensure that new interventions effectively enhance social competencies without intensifying affective problems, either by supporting the positive outcomes described in prior studies (2, 12, 25), or by addressing emerging emotional challenges during the intervention.

Several lessons emerged from this study that may guide future implementation efforts. Elements such as intervention duration, setting characteristics, the professionals involved, participant age and cognitive profile, as well as the assessment methods used for comorbid symptoms and social behaviors, proved to be critical factors. These aspects should be carefully considered when adapting and applying social skills programs in publicly funded mental health services.

### Limitations

First, a limitation of the study was the small sample size for each group and subgroup, which limits interpretations for a broader population and constraints statistical power in the effort to find significance in the tested hypothesis Additionally, the sample included a predominantly male group, which, although representative of clinical prevalence patterns in ASD, limits the generalizability of the results to girls with autism. Socioeconomic and ethnic background data were not analyzed in this pilot trial, but these factors should be carefully considered in future research to ensure broader applicability and equity in intervention outcomes.

Second, a significant limitation was related to the intensity and the program duration, where the total number of sessions, frequency, and/or total duration in time could make a significant difference in our tested hypothesis. Researchers reduced the number of sessions to half of the original twenty to make it available to a larger number of patients that met the initial inclusion criteria for the clinical program and reduce, as much as possible, the waiting time to begin the program.

Third, there were limitations related to measures. Social behaviors were evaluated with an observational instrument in the context of a social skills group, but the study did not include a caregiver scored measure of social interaction, which could have offered information about generalization of skills into other domains of life. However, parents burden is intense regarding participating in a trial so researchers aim to prevent drop-outs by lessening family questionnaires.

Fourth, this observational coding was conducted on a subgroup of participants whose video recordings met criteria for visibility and duration. Due to the use of static cameras in a large room where participants could move freely, some recordings did not adequately capture the child’s behavior and had to be excluded. Although comparisons showed no significant differences between the video-coded subgroup and the overall sample, the exclusion process could represent a potential source of selection bias.

Other limitations included resource constraints within the public healthcare setting where the study was implemented, that lead to an abbreviated cognitive assessment (WISC verbal IQ). As a result, detailed cognitive indices such as verbal comprehension, working memory, or processing speed could not be systematically reported, which limits the granularity of participant characterization and better understanding of the treatment response to targeted subgroups. Also, the lack of parent components and the lack of an independent rater or external therapist to evaluate the “implementation” fidelity, which could be biased by the team leader’s supervision across sessions or age group. Additionally, a waitlist-controlled design was used, and the study did not include follow-up assessments to evaluate maintenance of treatment effects due to the community setting where research was performed.

Finally, the quantity of data collected from the observational methodology was constrained by technological resources (e.g., recording angles) and other factors that limited the sample and the data collection itself, directly affecting the available sample with reliable observational and coded data, in turn affecting the statistical power for the primary objective. No correction for multiple comparisons was applied, which may increase the risk of Type I error. However, this decision was made to preserve statistical power and reduce the risk of Type II error, which is a relevant concern in pilot studies with small sample sizes. Future studies with larger samples should consider multiplicity adjustments to confirm these findings.

## Conclusions

Despite its limitations, this study offers initial findings contributing to the growing evidence base for implementing social skills interventions in publicly funded clinical services. Adapting evidence-based interventions into formats feasible for publicly funded health care services and conducting research to evaluate their effect is highly challenging. However, this research holds important clinical implications, highlighting the need to balance feasibility and implementation fidelity in contexts where resources are constrained. Following this ethical rationale, community services should strive to implement interventions that, while feasible, still meet efficacy standards and show measurable benefits for participants.

The findings of this study align with prior research documenting similar implementation challenges in publicly funded settings (35, 43). For instance, limited resources often require low-intensity programs (reduced number of sessions and limited duration), which allow access to a larger number of patients and respond to the needs of publicly funded hospital services. Developing brief and scalable programs that demonstrate measurable changes is crucial.

The results of the present randomized controlled pilot efficacy trial indicate that this structured social skills program led to measurable improvements in social skills (eye-contact and functional communication) in the children subgroup and participants with higher verbal IQ, as observed through observational behavioral methodology. Observational methodology might be particularly suitable for observing social behaviors during social skills intervention (40, 53) as a proximal measurement of targeted intervention and has been highlighted as a key tool to detect subtle changes in short-duration programs (65). Observational methodology allows the study of spontaneous behaviors and provides more detailed and accurate information about social behavior that questionnaires might not adequately capture (2, 9, 38, 39).

Similarly, results showed no adverse effects and a meaningful reduction in internalizing problems in children subgroup. In contrast, no significant improvements in social skills were observed in the adolescent group, and an increase in anxiety symptoms was reported in this subgroup following the intervention. These findings support the efficacy and beneficial effects of the intervention in enhancing social competencies and mental health outcomes for autistic individuals with higher verbal abilities, particularly in childhood.

While no overall adverse effects were found in the full sample, a significant increase in anxiety was identified in the adolescent subgroup. This suggests that while the program is broadly beneficial, it may present specific challenges for adolescents that need to be addressed.

### Recommendations for clinical practice

#### Monitor anxiety levels in adolescents

Clinicians implementing social skills interventions should pay close attention to anxiety levels in adolescent participants. Regular monitoring and assessment of anxiety can help identify any emerging issues early and allow for timely intervention. Assigning a co-therapist to each adolescent may increase the probability of detecting anxiety symptoms and respond to the emotional needs during sessions by adjusting specific treatment components. It is also essential to clinically evaluate the cause of anxiety in each individual to act preventively during the Social Skills intervention.

#### Tailor interventions to age groups

Given the significant effect on anxiety observed in adolescents, it is recommended that social skills programs be tailored to meet the specific needs of different age groups. Adolescents may require additional support and modifications to the program to prevent increases in anxiety. The program duration might be inappropriate for this age group, which may need a more extended adaptation period.

#### Incorporate coping strategies

Incorporating coping strategies and anxiety management techniques into the social skills curriculum, especially for adolescents. Teaching relaxation techniques, mindfulness, and stress reduction methods can help mitigate the risk of increased anxiety associated with a social skills program.

#### Continuous evaluation

Implement ongoing evaluations of the program’s effectiveness and impact on mental health across different age groups. Collecting regular feedback from participants can inform adjustments to better address the needs of all participants.

#### Collaborative approach

Encourage collaboration between clinicians, educators, and parents to ensure a comprehensive support system for participants. This team approach can help monitor and address any issues that arise during the intervention.

Incorporating these recommendations can help maximize the effectiveness of social skills interventions while minimizing potential risks, particularly for adolescents, and promote better outcomes for autistic individuals.

## Data Availability

The raw data supporting the conclusions of this article will be made available by the authors, without undue reservation.
